# Increasing serotonin bioavailability alters gene expression in peripheral leukocytes and lymphoid tissues of dairy calves

**DOI:** 10.1038/s41598-020-66326-w

**Published:** 2020-06-16

**Authors:** M. G. Marrero, S. L. Field, A. L. Skibiel, B. Dado-Senn, J. P. Driver, J. Laporta

**Affiliations:** 1 Department of Animal Sciences, University of Florida, Florida, USA; 2 Present Address: Department of Animal and Veterinary Science, University of Idaho, Idaho, USA

**Keywords:** Physiology, Immunology

## Abstract

Dairy calves are born with a naïve immune system, making the pre-weaning phase a critical window for immune development. In the U.S., 40–60% of dairy farms feed milk replacer to pre-weaned calves, which are devoid of bioactive factors with immunological roles. Serotonin is a bioactive factor with immunoregulatory properties naturally produced by the calf and present in milk. Human and rodent immune cells express the serotonin machinery, but little is known about the role of serotonin in the bovine immune system. Supplementing milk replacer with 5-hydroxytryptophan (serotonin precursor) or fluoxetine (reuptake inhibitor) increases serotonin bioavailability. We hypothesized that increased serotonin bioavailability promotes serotonergic signaling and modulates the expression of immune related genes in peripheral leukocytes and immune-related tissues of dairy calves. The present experiment targeted candidate genes involved in serotonin production, metabolism, transport, signaling and immune regulation. We established that bovine peripheral leukocytes express all known serotonin receptors, and can synthesize, uptake and degrade serotonin due to the expression of serotonin metabolism-related genes. Indeed, we showed that increasing serotonin bioavailability alters gene expression of serotonin receptors and immune-related genes. Further research will determine whether manipulation of the serotonin pathway could be a feasible approach to bolster dairy calves’ immune system.

## Introduction

Dairy calves are born with a naïve immune system. Feeding newborn calves high quality colostrum is a practice readily implemented on most U.S. dairy farms^1,2^. While colostrum is important for calf immune protection and survival, the remaining pre-weaning phase consists of a liquid diet of either whole milk or milk replacer. In the U.S., 40–60% of dairy farms feed milk replacers to pre-weaned dairy calves^3^. Dairy calves’ adaptive immune system develops gradually, and the pre-weaning phase has been shown to be critical for immune system development and maturation^4^. Emerging data demonstrate that milk not only delivers nutrients, but also primes the newborn’s growth and development through delivery of bioactive factors^5,6^. Although milk replacer formulation has improved over the years, it still lacks bioactive components naturally present in milk that could aid in the development of the dairy calf immune system. Therefore, there is a need to explore novel bioactive factors that when added to milk replacers can enhance dairy calf immune development.

Serotonin is a bioactive factor with immunoregulatory properties^7–12^ that is present in cow milk and is also endogenously synthesized by the calf^13–15^. However, little is known about the immunologic role of serotonin in milk or in cattle. Serotonin is derived from the conversion of L-tryptophan to 5-hydroxytryptophan (5-HTP) by the rate limiting enzyme tryptophan hydroxylase (TPH1, in peripheral tissues, and TPH2, in the brain), which is subsequently converted to serotonin by the aromatic amino acid decarboxylase enzyme (AADC/*DDC*)^16^. There are 7 serotonin receptor families with more than 10 G-protein coupled receptor (GPCR) subtypes and 3 ion-gated channel receptor subtypes^17^. Depending on which serotonin receptor subtype (G_s_, _q/11_ or _i/o_) is activated, signaling cascades including adenylyl cyclase (AC), protein kinase C (PKC), inositol trisphosphate (IP3) and mitogen and extracellular signal regulated kinase (MERK) are activated^18^ to modulate the activity of proteins or to regulate gene transcription. Serotonin action is terminated by the serotonin transporter (SERT), which removes circulating serotonin from the extracellular space to be recycled or degraded by monoamine oxidase (MAO).

Peripheral serotonin (close to 95% of total serotonin in the body) is primarily synthesized by the enterochromaffin cells in the gut and is involved in the regulation of many physiological functions^14,19–23^, including immune modulation. Peripheral serotonin is mainly stored and transported by blood platelets^24^, the major source of serotonin for circulating immune cells and organs. Several studies support the immunomodulatory role of peripheral serotonin in the rodent and human model. For instance, platelet-derived serotonin in mice promotes neutrophil recruitment to inflammation sites by increasing L-selectin expression and enhancing endothelial interactions^25^. Research in humans and rodents show that different immune cells express one or multiple components of the serotonergic signaling pathway machinery (i.e., receptors, TPH1 and/or SERT, MAO^8,9,26,27^), indicating their capacity to synthesize, metabolize, respond to, and/or transport serotonin^11,28,29^. Dendritic cells (DCs) do not express *TPH1*, however upon activation they increase *SERT* expression which allows them to take up serotonin from circulation^8^. Activation of murine T lymphocytes increases *TPH1* expression and hence, endogenous serotonin production. This serotonin then acts as an autocrine-paracrine cytokine to enhance T cell proliferation or is taken up by circulating cells (i.e., DCs and platelets)^8,9^. Furthermore, in mice, serotonin can attract mast cells, which express both *TPH1* and *SERT*^11^, to inflammation sites^30^. Studies using *TPH1* knockout mice (lacking peripheral serotonin) show reduced macrophage infiltration and lower proinflammatory cytokine production (i.e. IL-1β and -6) compared to wild type mice^31^. Dendritic cells of *TPH1* knockout mice produce less IL-12 following a 24 h *in vitro* lipopolysaccharide (LPS) challenge compared to wild type mice^32^. It has also been shown that isolated monocytes incubated with LPS secrete more cytokines when serotonin is present^26^.

Serotonin has been shown to regulates physiological functions that are relevant to lactation performance including metabolic status, milk synthesis and calcium regulation^15,23,33^. However, studies exploring serotonin’s immunomodulatory role are limited in the bovine. One study showed that supplementation of 5-hydroxytryptophan to newborn calves for 15 days increased blood mRNA abundance of genes related to innate and adaptive immunity, including nuclear factor kappa beta, chemokine C-C motif ligand 5, cyclooxygenase-2 and interleukin 1 beta^34^. However, a more thorough characterization of the bovine serotonergic pathway and its ability to modulate immunity is lacking. Herein, we characterize the expression profile of genes involved in serotonin synthesis, metabolism and signaling, and its impact on cytokine expression in leukocytes and lymphoid tissues of dairy calves supplemented with 5-hydroxytryptophan, the serotonin precursor, or fluoxetine, a selective serotonin reuptake inhibitor (SSRI). We hypothesized that increased cell and tissue serotonin bioavailability will promote the expression of genes involved in serotonergic machinery and signaling, and positively modulate the expression of immune genes in peripheral leukocytes, spleen, thymus and popliteal lymph node of pre-weaned dairy calves.

## Results

### Effects of FLX and 5-HTP supplementation on white blood cells counts and subfractions

No differences were observed for total WBC (count/μL) among treatment groups before or after 10 days of FLX or 5-HTP supplementation (*P* > 0.19; Table 1). Likewise, WBC subfractions (count/μL) including neutrophils, eosinophils, basophils, monocytes and lymphocytes were not different among treatment groups before or after 10 days of supplementation (*P* > 0.47; Table 1).

**Table 1:** Circulating white blood cells (neutrophils, lymphocytes, monocytes, eosinophils, and basophils) in dairy calves with increased serotonin bioavailability.

WBC count	Treatments^a^			*P-*value^b^	
	CON	5-HTP	FLX	5-HTP *vs*. CON	FLX *vs*. CON
Neutrophils, 10^3^/μL	3.69 ± 0.62	3.96 ± 0.67	3.69 ± 0.62	0.78	0.99
Monocytes, 10^3^/μL	1.08 ± 0.11	1.19 ± 0.12	0.97 ± 0.11	0.51	0.48
Lymphocytes, 10^3^/μL	4.37 ± 0.23	4.79 ± 0.24	4.79 ± 0.23	0.22	0.48
Eosinophils, 10^3^/μL	0.06 ± 0.02	0.06 ± 0.22	0.09 ± 0.02	0.96	0.42
Basophils, 10^3^/μL	0.01 ± 0.02	0.05 ± 0.03	0.03 ± 0.02	0.26	0.71

^a^Oral supplementation of milk replacer with saline (control; n = 8), fluoxetine (40 mg/d; n = 8) or 5-hydroxytryptophan (5-HTP, 90 mg/d; n = 8) to Holstein dairy calves for 10 consecutive days.

^b^Statistical significance declared at *P*-value ≤ 0.05 and tendencies at 0.05 < *P* ≤ 0.10.

### Effects of 5-HTP on peripheral leukocyte gene expression

#### Serotonin synthesis, metabolism, and downstream pathways

**Figure 1:**
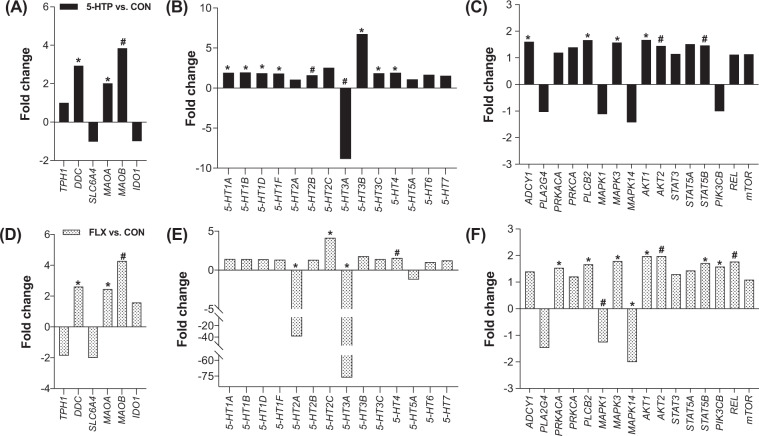
Gene expression in peripheral leukocytes of pre-weaned dairy calves after a 10-day oral supplementation of 5-hydroxytryptophan (5-HTP, 90 mg/d; n = 8), fluoxetine (FLX, 40 mg/d; n = 8) or saline (CON; n = 8). Gene expression is reported as fold change (2^−ΔΔCt^) relative to CON saline-supplemented group. Gene expression fold change of (**A**) genes related to serotonin synthesis and metabolism, (**B**) serotonin receptors, and (**C**) their downstream pathways after 10 days of 5-HTP oral supplementation. Gene expression fold change of (**D**) genes related to serotonin synthesis and metabolism, (**E**) serotonin receptors and (**F**) their downstream pathways. Black bars denote 5-HTP *vs*. CON gene expression fold change, and dotted bars denote FLX *vs*. CON gene expression fold change. The negative inverse of fold-change values <1 was calculated for visual representation of negative fold changes. (*) indicate significant differences (*P* ≤ 0.05) and (#) indicate tendencies (0.05 < *P* ≤ 0.10) between CON and FLX or CON and 5-HTP treatments.

After 10 days of 5-HTP supplementation, peripheral leukocytes were isolated for gene expression analysis, reported as fold change relative to CON saline-supplemented group. Supplementation of 5-HTP upregulated or tended to upregulate genes involved in serotonin synthesis and metabolism, including *DDC* and *MAOA* (*P* < 0.03) and *MAOB* (*P* = 0.09), while *TPH1*, *SLC6A4* and *IDO1* gene expression was not affected (*P* > 0.94; Fig. 1A). Seven serotonin receptors were upregulated including 5*-HT1A, -1B, -1D, -1F, -3B, -3C* and *-4* (*P* < 0.04) while *5-HT2B* tended to be upregulated (*P* = 0.06; Fig. 1B). The 5-*HT3A* receptor subtype was downregulated more than 8-fold following 5-HTP supplementation (*P* = 0.06; Fig. 1B), whereas the expression of *5HT2A*, -*5A*, *-6* and *-7* remained unchanged (*P* > 0.13). Four genes downstream of serotonin GPCR signaling were significantly upregulated by 5-HTP supplementation, including *ADCY1, PLCB2, MAPK3* and *AKT1 (P* < 0.05*);* while *AKT2* and *STAT5B* tended to be upregulated (*P* < 0.10; Fig. 1C).

#### Clusters of differentiation and immune related genes

**Figure 2:**
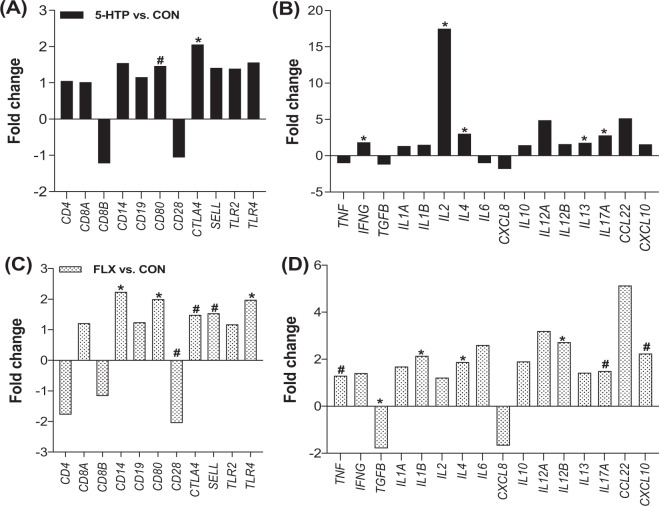
Gene expression in peripheral leukocytes of pre-weaned dairy calves after a 10-day oral supplementation of 5-hydroxytryptophan (5-HTP, 90 mg/d; n = 8), fluoxetine (FLX, 40 mg/d; n = 8) or control (CON; n = 8). Gene expression is reported as fold change (2^−ΔΔCt^) relative to CON saline-supplemented group. (**A**) Gene expression of immune surface markers and (**B**) cytokines after 10 days of 5-HTP oral supplementation. (**C**) Gene expression fold change of immune surface markers and (**D**) cytokines after 10 days of FLX oral supplementation. Black bars denote 5-HTP *vs*. CON gene expression fold change, and dotted bars denote FLX *vs*. CON gene expression fold change. The negative inverse of fold-change values <1 was calculated for visual representation of negative fold changes. (*) indicate significant differences (*P* ≤ 0.05) and (#) indicate tendencies (0.05 < *P* ≤ 0.10) between CON and FLX or CON and 5-HTP.

**Figure 3:**
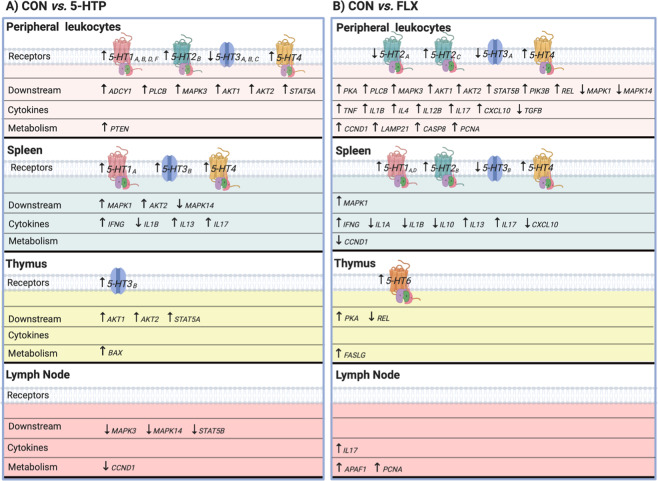
Summary of serotonin receptors, intracellular downstream signaling, cytokines and metabolism genes that were differentially expressed at the mRNA level in peripheral leukocytes (n = 8 per treatment) and lymphoid tissues (spleen, thymus and lymph node, n = 4 per treatment) of pre-weaned dairy calves after (**A**) 10-d 5-hydroxytryptophan oral supplementation (5-HTP, 90 mg/d) *vs*. 10-d saline oral supplementation (CON), and (**B**) 10-d Fluoxetine oral supplementation (FLX, 40 mg/d) *vs*. 10-d saline oral supplementation (CON). Summarized genes were (*P* ≤ 0.05) or tended (0.05 < *P* ≤ 0.10) to be differentially expressed between 5-HTP *vs*. CON or FLX *vs*. CON groups.

The expression of several immune related genes in peripheral leukocytes was modulated by 5-HTP supplementation. Specifically, *CTLA4* was upregulated (*P* < 0.006), and *CD80* tended to be upregulated compared to CON (*P* = 0.10; Fig. 2A). A significant upregulation of various cytokines including *IFNG, IL2, IL4, IL13*, and *IL17A* (*P* < 0.02) was observed, with *IL2* having the highest fold-change of 17.5 when compared to CON (Fig. 2B). Differentially expressed genes in peripheral leukocytes are summarized in Fig. 3A.

### Effects of FLX on peripheral leukocyte gene expression

#### Serotonin synthesis, metabolism and downstream pathways

After 10 days of FLX supplementation, peripheral leukocytes were isolated for gene expression analysis and reported as fold change relative to CON saline-supplemented group. Supplementation of FLX upregulated genes involved in serotonin synthesis and metabolism, including *DDC* and *MAOA* (*P* < 0.03) and tended to upregulate *MAOB* (*P* = 0.07), whereas *TPH1*, *SERT* and *IDO1* were not differentially expressed (*P* > 0.12; Fig. 1D). Fluoxetine supplementation upregulated the expression of *5-HT2C (P* = 0.03) and *5-HT4* (*P* = 0.08); while *5-HT2A* and *-3A* were significantly downregulated (>30-fold, *P* < 0.006; Fig. 1E). All other serotonin receptors remained unchanged (*P* > 0.12). Eight genes downstream of serotonin receptor signaling were upregulated including *PKA, PLCB2, MAPK3, AKT1, STAT5B* and *PIK3CB* (*P* < 0.03) or tended to be upregulated such as *AKT2* and *REL (P* < 0.10) by FLX supplementation (Fig. 1F). Meanwhile, *MAPK14* was significantly downregulated (*P* = 0.008) and *MAPK1* tended to be downregulated by FLX (*P* = 0.09, Fig. 1F).

#### Clusters of differentiation and immune related genes

The expression of various immune related genes in peripheral leukocytes was modulated by FLX supplementation. Specifically, FLX upregulated the gene expression of *CD14, CD80*, and *TLR4* (*P* < 0.045), *CTLA4*, and *SELL* (*P* < 0.10), while it downregulated *CD28* expression compared to CON (*P* < 0.10; Fig. 2C). Supplementation of FLX upregulated the gene expression of various cytokines, including *IL1B, IL4* and *IL12B* (*P* < 0.03), *TNF, IL17A* and *CXCL10* (*P* < 0.10; Fig. 2D). Only *TGFB* gene expression in peripheral leukocytes was significantly downregulated after the 10-d FLX supplementation (*P* = 0.04; Fig. 2D). Differentially expressed genes in peripheral leukocytes are summarized in Fig. 3B.

### Effects of 5-HTP on Thymus, Spleen, and Lymph Node Gene Expression

#### Serotonin synthesis, metabolism and downstream pathways

Supplementation of 5-HTP for 10 days did not affect serotonin metabolism related genes in spleen tissue compared to CON (*P* > 0.15; Supplementary Fig. S1A), but upregulated serotonin receptors *5-HT1A* and *-4* (*P* < 0.01) and tended to upregulate *5-HT3B* (*P* = 0.09; Supplementary Fig. S1B). In the spleen, *MAPK1* and *AKT2* tended to be upregulated, while *MAPK14* tended to be downregulated (*P* < 0.07; Supplementary Fig. S2A). In the popliteal lymph node, serotonin metabolism related enzyme *MAOB* tended to be upregulated (*P* = 0.06; Supplementary Fig. S1A), but no serotonin receptors were differentially expressed by 5-HTP (*P* > 0.11; Supplementary Fig. S1B). Additionally, in the lymph node, *MAPK3* and *STAT5B* tended to be downregulated (*P* > 0.08; Supplementary Fig. S2A). In the thymus, 5-HTP supplementation upregulated *DDC* (*P* = 0.04) and tended to downregulate *TPH1* (*P* = 0.07; Supplementary Fig. S1A), while it significantly upregulated the serotonin receptor *5-HT3B* (*P* = 0.02; Supplementary Fig. S1B). Thymus expression of genes downstream of serotonin receptors included upregulation of *AKT1* and *AKT2* (*P* < 0.043) and *STAT5A* (*P* = 0.06; Supplementary Fig. S2A). Differentially expressed genes in tissues are summarized in Fig. 3A.

#### Clusters of differentiation and immune related genes

The expression of several immune related genes in lymphoid tissues was altered by 5-HTP supplementation. In the spleen, 5-HTP supplementation upregulated the surface protein *CTLA4* (*P* = 0.01) and tended to downregulate the T cell surface marker, *CD8B* (*P* = 0.07; Supplementary Fig. S3A). Spleen expression of *IL17A* was upregulated (*P* = 0.04) and *IFNG* and *IL13* tended to be upregulated (*P* < 0.09) by 5-HTP supplementation; while *IL1B* cytokine was downregulated (P = 0.003; Supplementary Fig. S3B). In the popliteal lymph node, *CD14* was downregulated (*P* = 0.04; Supplementary Fig. S3A) but cytokine gene expression was not affected by 5-HTP supplementation (*P* > 0.16; Supplementary Fig. S3B). In the thymus, the surface protein, *CTLA4*, gene expression was upregulated (*P* = 0.025; Supplementary Fig. S3A), however cytokines were not differentially expressed after 5-HTP supplementation (*P* > 0.11; Supplementary Fig. S3B).

### Effects of FLX on Thymus, Spleen, and Lymph Node Gene Expression

#### Serotonin synthesis, metabolism and downstream pathways

In the spleen, FLX supplementation upregulated *SLC6A4* (*P* = 0.02) and *IDO1* (*P* = 0.07) and downregulated *TPH1* (*P* = 0.06; Supplementary Fig. S1C). Additionally, serotonin receptors *5-HT1A* and *-4* were upregulated (*P* < 0.04), *5-HT1D* and *-2B* tended to be upregulated (*P* < 0.08), while *5-HT3B* tended to be downregulated (*P* = 0.054; Supplementary Fig. S1D). Only *MAPK1* gene expression was upregulated by FLX (*P* = 0.03), while all other downstream serotonin receptor signaling genes remained unchanged in the spleen compared with CON calves (Supplementary Fig. S2B). In the popliteal lymph node, *REL* gene expression was downregulated following FLX supplementation (*P* = 0.002; Supplementary Fig. S2B). In the thymus, FLX tended to upregulate *5-HT6* receptor (*P* = 0.09) and the downstream *PKA* (*P* = 0.04), but the expression of genes related to serotonin metabolism or downstream pathways was similar to CON (*P* > 0.11; Supplementary Fig. S1D, S2B). Differentially expressed genes are summarized in Fig. 3B.

#### Clusters of differentiation and immune related genes

The expression of several immune related genes in lymphoid tissues was modulated by FLX supplementation. In the spleen, FLX upregulated the surface protein *CTLA4* (*P* = 0.03) and tended to downregulate *CD28* (*P* < 0.10; Supplementary Fig. S3C). The gene expression of *IFNG* and *IL17A* was upregulated (*P* < 0.04) and *IL13* tended to be upregulated (*P* = 0.08; Supplementary Fig. S3D). Meanwhile, the expression of *CXCL10* was downregulated (*P* = 0.001) and *IL1A*, *IL1B* and *IL10* tended to be downregulated (*P* < 0.08; Supplementary Fig. S3D). Fluoxetine supplementation upregulated the expression of surface protein *CTLA4* (*P* = 0.021; Supplementary Fig. S3C) in the thymus, however, cytokine genes were not differentially expressed (*P* > 0.11; Supplementary Fig. S3D). In the popliteal lymph node tissue, *IL17A* tended to be upregulated (*P* = 0.10; Supplementary Fig. S3D) but no other cytokine or surface markers genes were differentially expressed following FLX supplementation (*P* > 0.11; Supplementary Fig. S3C, S3D).

### Effects of 5-HTP and FLX on Proliferation, Apoptosis, and Cell Metabolism Genes

#### Peripheral leukocytes

The expression of genes related to cell proliferation, apoptosis, cell metabolism and cell cycle in peripheral leukocytes after 10-d of 5-hydroxytryptophan or fluoxetine supplementation was evaluated. Supplementation of 5-HTP upregulated *PTEN* (*P* = 0.005), whereas FLX upregulated *CCND1* (*P* = 0.02) and tended to upregulate *LAMP2*, *CASP8* and *PCNA* (*P* < 0.10; Supplementary Table S1).

#### Secondary lymphoid tissues

In the spleen, 5-HTP supplementation had no effect on cell metabolism gene expression (*P* > 0.12) whereas FLX supplementation downregulated *CCND1* (*P* = 0.04; data not shown). In the popliteal lymph node, 5-HTP supplementation tended to downregulate *CCND1* expression (*P* = 0.07), while FLX supplementation upregulated *APAF1* and *CASP8* (*P* < 0.04; data not shown) and tended to upregulate *FASLG* and *PCNA* (*P* < 0.10; data not shown). In the thymus, 5-HTP upregulated *BAX* (*P* = 0.01), while FLX upregulated *FASLG* (*P* = 0.03; data not shown).

## Discussion

The role of serotonin as an immunoregulatory molecule has been widely demonstrated in human and rodents^7–11,25,27^, however, evidence supporting its role in livestock species is lacking. We previously reported that increasing serotonin bioavailability in dairy calves is possible through the supplementation of 5-hydroxytryptophan or fluoxetine^35^. Herein, we report the effects of increased serotonin bioavailability on circulating WBC count and the gene expression of peripheral leukocytes and secondary lymphoid organs of dairy calves undergoing immune system maturation. To our knowledge, this is the first experiment to characterize how the serotonin axis regulates the bovine immune system.

In this experiment, WBC and WBC subfractions including neutrophil, monocyte, lymphocyte, eosinophil, and basophil counts were within the normal physiological ranges for growing dairy calves and similar among treatment groups before and after 10 days of treatment supplementation. This indicates that increasing serotonin bioavailability for 10 days did not significantly alter immune cell populations. Even though we did not see an increase in neutrophil counts, it is possible that serotonin is improving neutrophil function. For instance, human neutrophils cultured *in vitro* with high concentrations of serotonin have higher motility than neutrophils grown in low serotonin conditioned media^36^. Platelet expression of FcγRIIA, a receptor that recognizes immune complexes, plays a role in inflammation by activating neutrophils and enhancing endothelial vasodilatation^37^. Furthermore, neutrophils from wild type mice have been shown to have improved tissue infiltration compared to TPH1 knockout mice^25^. Thus, further *in vitro* experiments evaluating serotonin’s role in neutrophil motility and function (i.e. oxidative burst and phagocytic capacity) are needed in bovine.

For over 20 years, researchers have investigated the significance of serotonin receptors in human and murine immune cells and their possible implication in autoimmune diseases. In our experiment, the entire serotonergic machinery, including genes involved in serotonin synthesis, mechanism of action, and metabolism were expressed in the circulating leukocytes of all calves. This indicates that peripheral leukocytes of dairy calves can synthesize, metabolize, uptake and degrade serotonin. Supplementation of either 5-HTP or FLX increased serotonin bioavailability^35^ and upregulated several genes involved in serotonin machinery in peripheral leukocytes. The ubiquitous aromatic decarboxylase enzyme, *DDC*, that converts 5-HTP to serotonin, and monoamine oxidase enzyme, *MAOA*, that metabolizes serotonin, were significantly upregulated in the peripheral blood leukocytes of both 5-HTP and FLX fed calves. This suggests an overall increase of serotonin metabolism in immune cells of calves under supplementation.

Notably, supplementing 5-HTP upregulated the gene expression of 9 out of the 13 serotonin receptor subtypes evaluated in peripheral leukocytes compared to controls. Interestingly, all serotonin receptors from family 1 subtypes (5-HT1), including *-1A*, *-1B*, *-1D*, and *-1F* were significantly upregulated, suggesting a positive feedback loop to increase ligand binding. Serotonin receptor family 1 proteins couple mainly through G_i/o_ proteins to inhibit adenylyl cyclase in various cells and tissues and have high affinity towards serotonin^18^. Serotonin receptor 5-HT1 subtypes are expressed in various human and mouse immune cells including monocytes/macrophages, dendritic cells, neutrophils, mast cells, eosinophils, B cells and T cells^7^. Data in human and/or rodents shows that 5-HT1A receptor subtype modulates adhesion and chemotaxis of mast cells^30^ and enhances phagocytosis by murine macrophages^38^. Upregulation of serotonin receptors subtypes from family 1 in peripheral leukocytes after 5-HTP supplementation could support calves’ immune function by promoting adhesion and chemotaxis of mast cells, and/or enhancing phagocytosis. However, future functional studies targeting this specific receptor should be conducted in the bovine.

In this experiment, the *5-HT2B* receptor subtype was upregulated following 5-HTP supplementation. Activation signals among 5-HT2 family receptor subtypes are different, but mainly couple through G_q/11_ proteins. For instance, the 5-HT2A receptor subtype signals through the activation of PLC-β in tissues and cells, whereas the 45% homologous 5-HT2B receptor subtype signal through various phospholipases (i.e. PLC-β, PLA)^18^. This 5-HT2B receptor subtype has been widely studied in human dendritic cells (DCs) where it is reported to promote anti-inflammatory functions. Human monocytes cultured in the presence of serotonin, as well as IL-4 and granulocyte-macrophage colony-stimulating factor, differentiate into DCs with reduced expression of co-stimulatory molecules (i.e. CD86) which are needed for antigen-presenting cells (APC; i.e. DCs) and T cell cognate interactions^39^. Similarly, 5-HT2B receptor activation was found to downregulate monocyte derived DC expression of co-stimulatory molecules that activate naïve T cells, and possibly preventing inflammation by regulating both innate and adaptive immune systems^40^. Apart from *5-HT2B*, 5-HT2A expression is upregulated on activated CD4+ and CD8+ T cells^41^. Furthermore, 5-HT2A antagonist treatment inhibits T cell activation and diminishes IL-2 and interferon gamma (IFN-γ) production in a dose dependent manner^41^. Thus, it appears that 5-HT2A acts as a proinflammatory serotonin receptor whereas 5-HT2B acts as an immunosuppressive serotonin receptor.

Herein, both *IL2* and *IFNG* gene expression were upregulated even though *5-HT2A* was not differentially expressed. Interleukin-2 is an immunoregulatory cytokine, mainly produced by CD4+ T cells, which enhances T cell proliferation, regulates T helper cell differentiation^42^, and limits immune responses by enhancing T-regulatory cells function^43^. Moreover, IL-2 induces the transcription of IFN-γ in T cells^44^. Interferon gamma is an important inflammatory cytokine that induces maturation and licensing of APC that in turn recruit and prime T cells, and increases the expression of major histocompatibility complex^45^. Translation of IFN-γ by T cells skews B cells to enhance antibody production and induces isotype switching from IgM to IgG2a^46^. Upregulation of *IL2* and *IFNG* by 5-HTP supplementation, if translated to protein, could act to support adaptive immune responses. Further studies are warranted to confirm and determine the implications of these findings.

The serotonin receptors -*3B* and -*3C*, and *-4* were upregulated following 5-HTP supplementation. The 5-HT3B receptor subtype is a ligand-gated ion channel^47^ and is 41% homologous to the 5-HT3A receptor^48^. This receptor has been linked to nausea in patients undergoing chemotherapy^49^. To our knowledge the function of 5-HT3B receptor subtype has not been linked to immunity, probably because 5-HT3B can be expressed as a heteromeric receptor, 5-HT3A/B, with properties differing from those of 5-HT3A receptor subtype^50^.

Gene expression of key signaling molecules downstream of serotonin receptors, including *ADCY1, PLCB2, MAPK3* and *AKT*, was upregulated by 5-HTP supplementation. Adenyl cyclase (*ADCY1*) is a major downstream signaling gene for 5-HT4, -6 and -7 following G_s_ coupling activation^18^. Yet, in some cell types, activation of 5-HT1A receptor inhibits *ADCY1*^51^. Since both 5-HT1 and -4 receptor families were upregulated in our experiment we cannot determine which specific serotonin receptor subtype might be upregulating the expression of *ADCY1* intracellularly. Furthermore, both *PLCB2* and *5-HT2B* were differentially expressed and PLCB2 is known to be the major signaling downstream molecule for 5-HT2B^52^. Yet, further investigation is needed to characterize the effects of specific serotonin receptors and downstream pathways on specific circulating immune cells in the bovine.

The gene expression of clusters of differentiation *CD4*, *CD8* and *CD14* in peripheral leukocytes were not affected by 5-HTP supplementation, suggesting that increased serotonin bioavailability had no effect on immune cell concentrations. However, serotonin has been linked to the migration of specific immune cell types. For instance, Müller *et al*.^53^ reported that 5-HT1B receptor induces the migration of human immature DCs. Additionally, human DCs have been shown to secret IL-1 $\beta$ after activation of receptors 5-HT3, -4 and -7^54^. Interestingly, in our experiment, peripheral blood leukocytes had greater expression of serotonin receptor subtypes *5-HT3B*, *-3C* and *-4*, although upregulation of *IL1B* was not observed. Hernandez-Castellano *et al*.^55^ supplemented 5-HTP to newborn dairy calves for 15 days and reported an upregulation of both IL-1 $\beta$ and nuclear factor kappa beta (NF-$\kappa \beta$) genes in blood but no differences in IgG production were observed. In our experiment, *REL* (NF-$\kappa \beta$ subunit) was not affected by 5-HTP supplementation, however discrepancies between Hernandez-Castellano’s and our results could be attributed primarily to calf age linked to different facets of immune system development.

To explore serotonin’s role on immune system activation, we also evaluated the gene expression of surface molecules and cytokines. Supplementation of 5-HTP for 10 days tended to upregulate *CD80* gene expression and upregulated *CTLA4*, its preferential binding partner^56^. The cytotoxic T-lymphocyte-associated protein 4 (CTLA-4) is a protein expressed by regulatory T cells and activated T cells that exerts negative feedback to diminish T cell responses, whereas *CD80* is a costimulatory molecule expressed on APC^57^. As previously mentioned, 5-HTP supplementation upregulated *IL2* gene expression, which plays an important role in T-helper cell proliferation and survival, and T-regulatory cell activation^42^. Our findings suggest that greater serotonin bioavailability might activate serotonin receptors on immune cells, promoting IL-2 production, T cell activation, and eventually *CTLA4* expression to prevent excessive T cell activation^42,43^. Thus, we propose that the serotonin axis may play a role in balancing the immune system by promoting protective immune responses and preventing potentially dangerous inflammation.

Fluoxetine oral supplementation increases serotonin bioavailability by blocking SERT^58^. Fluoxetine supplementation downregulated *5-HT2A* and *-3A* receptor subtypes by more than 20-fold in peripheral leukocytes. Contradictory findings have been reported linking the use of SSRIs to 5-HT2A downregulation in the rodent frontal cortex^59^. Nevertheless, the effects and implications of the downregulation of 5-HT2A receptor by SSRIs in the immune system remain unknown^59–61^. Monocytes and T cells express the 5-HT3A receptor subtype^62^, as well as naïve and activated B cells, predominantly by differentiating B cells at the germinal centers of lymph nodes^63^. Interestingly, the use of the 5-HT3A antagonist, tropisetron, inhibits T cell activation and production of IL-2^64^. In 1994, Fan^65^ reported that 5-HT3 receptor is a target of fluoxetine, which in turn decreases serotonin influx into the cell. Moreover, fluoxetine blocks 5-HT3 receptors by interactions during both open and closed channel states, although the clinical relevance of this effect is still unknown^66^. Thus, further research is needed to understand 5-HT3B downregulation by fluoxetine supplementation.

The thymus is a central lymphoid organ where T cells develop, while the spleen and lymph nodes are important secondary lymphoid organs where immune responses are generated^67^. Therefore, we sought to explore the effects of increased serotonin bioavailability in these tissues. Similar to peripheral leukocytes, all tissues, independent of treatment supplementation, expressed the serotonergic machinery indicating that serotonin could be playing a role in the development and deployment of adaptive immune responses. We demonstrated that increased serotonin bioavailability exerts a less pronounced effect in these tissues compared to peripheral leukocytes, at least at the mRNA level. However, it is important to mention that tissue data was collected from a subset of animals (n = 4/treatment) euthanized after 10 days of treatment supplementation and that the statistical power to detect significant differences in tissues was 66%. Nevertheless, it is notable that we observed a significant downregulation of *CD14* in the popliteal lymph node after 5-HTP supplementation and FLX tended to downregulate *CD8B* in spleen tissue, while both treatments upregulated *CTLA4* in the thymus and spleen tissues. *CD8* downregulation has been shown to occur when CD8 effector T cells are switching functions^68^. There is limited data exploring the effect of serotonin on thymus, lymph node, and/or spleen gene expression, thus, these results are novel and warrant deeper investigation.

The present experiment targeted candidate genes involved in serotonin production, metabolism, transport, signaling and immune regulation. We established that bovine peripheral blood leukocytes and immune tissues express components of the serotonin signaling pathway, including *TPH1*, *SERT*, *DDC*, *MAO* and serotonin receptors. This indicates that these cells and tissues have the potential to synthesize, transport, respond to and/or degrade serotonin. We demonstrated that at the mRNA level, increased serotonin bioavailability exerts a pronounced immunomodulatory response, particularly in peripheral leukocytes and spleen tissue. Indeed, specific serotonin receptors and cytokines were differentially expressed upon 5-HTP or FLX supplementation, which could potentially influence the developmental trajectory and maturation of immune cells in dairy calves at a young age. Differences in the modulatory effects of 5-HTP and FLX in the peripheral immune system could be attributed to intrinsic differences in their molecular mechanism of action. Indeed, we previously reported that 5-HTP calves had greater circulating serotonin concentrations when compared to fluoxetine calves^35^, thus we hypothesize that greater serotonin bioavailability exerts different effects. While promising correlations between serotonin and the immune system exist in other species, and now in the bovine at the transcriptional level, it is currently unknown whether promoting serotonin translates into both innate and adaptive immune system orchestration. Ultimately, the fact that 5-HTP is a biogenic modified amino acid whereas fluoxetine is a synthetic drug should be taken into consideration when developing strategies to enhance livestock health and development.

## Methods

### Animals and experimental design

All methods and procedures performed in this study were

carried out in accordance with relevant guidelines and regulations approved by the Institutional Animal Care and Use Committee at the University of Florida (protocol # 201709851). Experimental design and treatments are described in detail by Marrero *et al*.^35^. Briefly, Holstein bull calves (n = 24, 18 ± 2 d of age, 47 ± 3.2 kg) were assigned to one of three treatments in a complete randomized block design (8 pens, 2.3 m x 2.5 m; n = 3 per pen, one of each treatment). Calves received 4 L of milk replacer (Southeast Milk Inc, Okeechobee, FL) at 0700 h and 1700 h. Treatments were administered once daily (0700 h feeding) by supplementing milk replacer with 5-hydroxytryptophan (**5-HTP**, 90 mg/d, n = 8, Sigma, St. Louis, MO, USA; #H9772), fluoxetine (**FLX**, 40 mg/d, n = 8, Spectrum Chemical, Gardena, CA, USA; #F1200) or saline (**CON**, n = 8) for 10 consecutive days. Treatment was applied individually to each calf. Supplementing 5-HTP and FLX increases serotonin bioavailability by different mechanisms: exogenous 5-HTP bypasses the rate liming enzyme *TPH1*, allowing its conversion to serotonin by AADC^69^, whereas FLX binds to *SERT* inhibiting endogenous serotonin reuptake within the cell^58,70^.

### Hematology analysis

Whole blood samples were collected from the jugular vein before (d0) and on the 10^th^ day of treatment supplementation 4 h after 0700 h feeding in tubes containing K2 EDTA (BD, Franklin Lakes, NJ, USA, #368047). Within 2 h of collection, blood samples were analyzed for hematology parameters including white blood cells (WBC), neutrophil, lymphocyte, monocyte, eosinophil and basophil count/μL using the Idexx ProCyte Dx analyzer (IDEEX Laboratories Inc., Westbrook, ME).

### Peripheral blood mononuclear cell isolation

Blood samples were collected from the jugular vein on the 10^th^ day of treatment supplementation 4 h after the 0700 h feeding using heparin blood collection tubes (BD, Franklin Lakes, NJ, USA, # 366430) and kept on ice until laboratory arrival. Blood was centrifuged at 1,200 g for 18 min at 20 °C, plasma layer was discarded, and peripheral leukocytes (i.e., buffy coat) was transferred to a 15 mL conical tube. To lyse residual red blood cells, a hypotonic solution (lyse buffer:1.5 g Na2HPO4 (Fisher; #BP332-1) and 0.3 g NaH2PO4 (Fisher; #BP329-1) at pH 7.2) was used. To restore cells, restore buffer was used (27 g NaCl (Fisher cat. #BP358-1) added to the lyse buffer solution and adjusted to pH 7.2). To each sample, 8 mL of lyse buffer and 4 mL of restore buffer were added. Tubes were centrifuged at 650 g for 5 min at 4 °C to form a pellet. Pellets were resuspended in 200 µL of RNAlater (Invitrogen, Carlsbad, CA, USA; #AM7021) and stored at -80 °C until RNA extraction.

### Euthanasia and tissue collection

After the 10^th^ d of treatment supplementation, 4 calves per treatment (n = 4 pens) were euthanized at the University of Florida abattoir. Calves were sedated by intravenous administration of 0.2 mg/kg xylazine and euthanized using a captive bolt pistol followed by jugular exsanguination. Spleen, popliteal lymph node and thymus tissues (approximately 1 g each) were harvested, rinsed in sterile PBS, transferred to a cryotube containing RNAlater and stored at −80 °C until RNA extraction.

### Peripheral Leukocyte and Tissue RNA Extraction

Peripheral leukocytes resuspended in RNAlater were centrifuged at 650 g for 10 min at 4 °C to reform the pellet and RNAlater was removed. For tissues, RNA was extracted from 60 mg each of spleen, popliteal lymph node and thymus. Each sample was placed in 1 mL of QIAzol Lysis reagent (Qiagen, cat. #79306) and homogenized using a tissue homogenizer (Tissue Master 125, Omni International, GA, USA). A commercial RNA extraction kit (RNeasy Plus Universal Mini Kit, Qiagen, cat. #73404) was used according to the manufacturer’s instructions. RNA concentration and quality were determined using a NanoDrop (NanoDrop Spectrophotometer, Thermo Scientific, USA; #ND-2000). RNA samples were stored at −80 °C until gene expression analysis.

### Primer design, validation and, gene expression analysis

We quantified the expression of genes related to serotonin machinery and signaling (i.e., synthesis and metabolism, serotonin receptors, and downstream pathways), immune-related genes (i.e., cytokines), and genes involved in metabolic and cellular processes (i.e., apoptosis, cell cycle, among others) in peripheral leukocytes, spleen, thymus and popliteal lymph node tissues. For this, high-throughput Multiplex RT-qPCR BioMark Dynamic Array Integrated Fluidic Circuits (IFCs) was used (Fluidigm Corporation, South San Francisco, CA). Briefly, 96 primers targeting 91 genes of interest, 4 reference genes (*ACTB*, *GADPH*, *RSP9* and *HPRT1*), and one reference structural gene were assayed (see Supplementary Table S1). An initial quantification run was performed for primer validation using an 8-point, two-fold dilution series (in triplicate) using RNA pools per tissue of interest. The linearity between RNA quantity and cycle threshold (Ct) was tested and efficiency of amplification was calculated. Primers were considered validated if they passed 5 points with an efficiency of 0.8–1.3 and an R^2^ ≥ 0.92. Specificity of amplification for each primer pair was evaluated by plotting the dissociation-characteristics of double-stranded DNA. A single peak following melt curve analysis indicated a pure, single amplicon. For gene expression of immune-related tissues and peripheral leukocytes, RNA was diluted to 5–15 ng/μL. All samples were normalized to 256 pg RNA and transferred to the IFC plate with the primer-probe sets. All nanoliter reactions were performed as per manufacturer’s recommendations using the following thermal protocol: 95 °C for 1 min, followed by 30 cycles of 96 °C for 5 s and 60 °C for 20 s. The software, Fluidigm Real-Time PCR Analysis, was used to calculate Ct values for all 96 genes for each sample. Non-detectable expression was set at a Ct of 26 for spleen tissue and 28 for WBC, popliteal lymph node, and thymus tissues. The geometric mean of the four reference genes was calculated for each sample and used to normalize Ct values of genes of interest. Normalized gene expression (ΔCt) was used for statistical analysis.

### Statistical analysis

Data were analyzed using linear models in R programming 3.5.1 (R Foundation for Statistical Computing; Vienna, Austria). Variables analyzed were white blood cell counts and ΔCt of genes evaluated by qPCR. The model included pen and treatment (CON, 5-HTP or FLX) as fixed effects. For gene expression analysis, the estimates of the model (ΔΔCt) for each gene were used to calculate fold change relative to CON (i.e. 5-HTP *vs*. CON or FLX *vs*. CON), using the 2^-ΔΔCt^ method^71^. The negative inverse of fold-change values <1 was calculated for visual representation of negative fold changes. Homogeneity of variance and normality of residuals were evaluated by plotting and influential points were detected using Cook’s distance test. Peripheral leukocyte gene expression ΔCt averages for CON, 5-HTP and FLX, and fold changes and *P* values for 5-HTP *vs*. CON and FLX *vs*. CON can be found in Supplementary Table S2. Statistical significance was declared at *P* ≤ 0.05 and tendencies at 0.05 < *P* ≤ 0.10.

## Supplementary information

Supplementary Information.

## Data Availability

All data generated or analyzed during this study are included in this published article [and its supplementary figures and tables online].
