# Supplementary material for: Increasing serotonin bioavailability alters gene expression in peripheral leukocytes and lymphoid tissues of dairy calves
Source: Sci Rep. 2020 Jun 16;10:9712. doi: 10.1038/s41598-020-66326-w (PMC7297988; doi:10.1038/s41598-020-66326-w)
Supplement: Supplementary file 1 — Supplementary Information. [file 41598_2020_66326_MOESM1_ESM.docx]

**Increasing serotonin bioavailability alters gene expression in peripheral leukocytes and lymphoid tissues of dairy calves**

Marrero M.G. ^1^, Field S.L. ^1^, Skibiel A.L. ^1&^, Dado-Senn B. ^1^, Driver J.P.^1^ and Laporta J. ^1^*

^1^ Department of Animal Sciences, University of Florida, USA

* Corresponding author: J. Laporta, [jlaporta@ufl.edu](mailto:jlaporta@ufl.edu)

**Supplementary Figure S1.** Gene expression in lymphoid tissues of pre-weaned dairy calves after a 10-day oral supplementation of 5-hydroxytryptophan (5-HTP, 90 mg/d; n = 4), fluoxetine (FLX, 40 mg/d; n = 4) or saline (CON; n = 4). Gene expression is reported as fold change (2^-ΔΔCt^) relative to CON. Fold change of genes involved in serotonin synthesis and metabolism and serotonin receptors in spleen, popliteal lymph node and thymus after 10 days of 5-HTP (A, B) or FLX (C, D) oral supplementation. Black bars denote spleen, gray bars denote lymph node, and white bars denote thymus gene expression fold change relative to CON. The negative inverse of fold-change values < 1 was calculated for visual representation of negative fold changes. (*) indicate significant differences (*P* ≤ 0.05) and (#) indicate tendencies (0.05 < *P* ≤ 0.10) between CON *vs*. FLX or CON *vs*. 5-HTP groups.

**Supplementary Figure S2.** Gene expression in lymphoid tissues of pre-weaned dairy calves after a 10-day oral supplementation of 5-hydroxytryptophan (5-HTP, 90 mg/d; n = 4), fluoxetine (FLX, 40 mg/d; n = 4) or saline (CON; n = 4). Gene expression is reported as fold change (2^-ΔΔCt^) relative to CON. Fold change of genes involved in serotonin receptor downstream pathways in spleen, popliteal lymph node and thymus after 10 days of 5-HTP (A) or FLX (B) oral supplementation. Black bars denote spleen, gray bars denote lymph node, and white bars denote thymus gene expression fold change relative to CON. The negative inverse of fold-change values < 1 was calculated for visual representation of negative fold changes. (*) indicate significant differences (*P* ≤ 0.05) and (#) indicate tendencies (0.05 < *P* ≤ 0.10) between CON *vs*. FLX or CON *vs*. 5-HTP groups.

**Supplementary Figure S3.** Gene expression in lymphoid tissues of pre-weaned dairy calves after a 10-day oral supplementation of 5-hydroxytryptophan (5-HTP, 90 mg/d; n = 4), fluoxetine (FLX, 40 mg/d; n = 4) or saline (CON; n = 4). Gene expression is reported as fold change (2^-ΔΔCt^) relative to CON. Fold change of gene expression of immune surface markers and cytokines in spleen, popliteal lymph node and thymus after 10 days of 5-HTP (A, B) or FLX (C, D) oral supplementation. Black bars denote spleen, gray bars denote lymph node, and white bars denote thymus gene expression fold change relative to CON. The negative inverse of fold-change values < 1 was calculated for visual representation of negative fold changes. (*) indicate significant differences (*P* ≤ 0.05) and (#) indicate tendencies (0.05 < *P* ≤ 0.10) between CON *vs*. FLX or CON *vs*. 5-HTP groups.

**Supplementary Table S1.** Gene symbols and name of 95 genes tested.

|  | **Genes** | **Gene name** |
| --- | --- | --- |
| 1 | *5-HT1A* | Serotonin receptor 1A |
| 2 | *5-HT1B* | Serotonin receptor 1B |
| 3 | *5-HT1D* | Serotonin receptor 1D |
| 4 | *5-HT1F* | Serotonin receptor 1F |
| 5 | *5-HT2A* | Serotonin receptor 2A |
| 6 | *5-HT2B* | Serotonin receptor 2B |
| 7 | *5-HT2C* | Serotonin receptor 2C |
| 8 | *5-HT3A* | Serotonin receptor 3A |
| 9 | *5-HT3B* | Serotonin receptor 3B |
| 10 | *5-HT3C* | Serotonin receptor 3C |
| 11 | *5-HT4* | Serotonin receptor 4 |
| 12 | *5-HT5A* | Serotonin receptor 5A |
| 13 | *5-HT6* | Serotonin receptor 6 |
| 14 | *5-HT7* | Serotonin receptor 7 |
| 15 | *DDC* | Bos taurus aromatic decarboxylase enzyme |
| 16 | *ACTB* | Bos taurus actin beta |
| 17 | *ADCY1* | Bos taurus denyl cyclase |
| 18 | *AKT1* | Bos taurus AKT serine/threonine kinase 1 |
| 19 | *AKT2* | Bos taurus AKT serine/threonine kinase 2 |
| 20 | *ALDH2* | Bos taurus aldehyde dehydrogenase 2 family member |
| 21 | *APAF1* | Bos taurus apoptotic peptidase activating factor 1 |
| 22 | *BAX* | Bos taurus BCL2 associated X, apoptosis regulator |
| 23 | *BCL2* | Bos taurus BCL2, apoptosis regulator |
| 24 | *CASP3* | Bos taurus caspase 3 |
| 25 | *CASP8* | Bos taurus caspase 8 |
| 26 | *CASP9* | Bos taurus caspase 8 |
| 27 | *CCL22* | Bos taurus C-C motif chemokine ligand 22 |
| 28 | *CCND1* | Bos taurus cyclin D1 |
| 29 | *CD14* | Bos taurus cluster of differentiation 14 |
| 30 | *CD19* | Bos taurus cluster of differentiation 19 |
| 31 | *CD28* | Bos taurus cluster of differentiation 28 |
| 32 | *CD4* | Bos taurus cluster of differentiation 4 |
| 33 | *CD44* | Bos taurus cluster of differentiation 44 |
| 34 | *CD80* | Bos taurus cluster of differentiation 80 |
| 35 | *CD8A* | Bos taurus cluster of differentiation 8A |
| 36 | *CD8B* | Bos taurus cluster of differentiation 8B |
| 37 | *CREB1* | Bos taurus cAMP responsive element binding protein 1 |
| 38 | *CTLA4* | Bos taurus cytotoxic T-lymphocyte associated protein 4 |
| 39 | *CXCL10* | Bos taurus C-X-C motif chemokine ligand 10 |
| 40 | *CXCL8* | Bos taurus C-X-C motif chemokine ligand 8 |
| 41 | *DGKA* | Bos taurus diacylglycerol kinase alpha |
| 42 | *FASLG* | Bos taurus Fas ligand |
| 43 | *GAPDH* | Bos taurus glyceraldehyde-3-phosphate dehydrogenase |
| 44 | *GSK3B* | Bos taurus glycogen synthase kinase 3 beta |
| 45 | *HP* | Bos taurus haptoglobin |
| 46 | *HPRT1* | Bos taurus hypoxanthine phosphoribosyltransferase 1 |
| 47 | *IDO1* | Bos taurus indoleamine 2,3-dioxygenase 1 |
| 48 | *IFNG* | Bos taurus interferon gamma |
| 49 | *IL10* | Bos taurus interleukin 10 |
| 50 | *IL12A* | Bos taurus interleukin 12A |
| 51 | *IL12B* | Bos taurus interleukin 12B |
| 52 | *IL13* | Bos taurus interleukin 13 |
| 53 | *IL17A* | Bos taurus interleukin 17A |
| 54 | *IL1A* | Bos taurus interleukin 1A |
| 55 | *IL1B* | Bos taurus interleukin 1B |
| 56 | *IL2* | Bos taurus interleukin 2 |
| 57 | *IL4* | Bos taurus interleukin 4 |
| 58 | *IL6* | Bos taurus interleukin 6 |
| 59 | *LAMP2* | Bos taurus lysosomal associated membrane protein 2 |
| 60 | *MAOA* | Bos taurus monoamine oxidase A |
| 61 | *MAOB* | Bos taurus monoamine oxidase B |
| 62 | *MAP1LC3B* | Bos taurus microtubule associated protein 1 light chain 3β |
| 63 | *MAP2K6/MEK* | Bos taurus mitogen-activated protein kinase kinase 6 |
| 64 | *MAPK1* | Bos taurus mitogen-activated protein kinase 1 |
| 65 | *MAPK14* | Bos taurus mitogen-activated protein kinase 14 |
| 66 | *MAPK3* | Bos taurus mitogen-activated protein kinase 3 |
| 67 | *MAP1LC3A* | Bos taurus microtubule associated protein 1 light chain 3 alpha |
| 68 | *MTOR* | Bos indicus mechanistic target of rapamycin |
| 69 | *REL* | Bos taurus REL proto-oncogene, NF-kB subunit |
| 70 | *NOS2* | Bos taurus nitric oxide synthase 2 |
| 71 | *PCNA* | Bos taurus proliferating cell nuclear antigen |
| 72 | *PDGFA* | Bos taurus platelet derived growth factor subunit A |
| 73 | *PI3KCB* | Bos taurus phosphatidylinositol-4,5-bisphosphate 3-kinase, catalytic subunit beta |
| 74 | *PLA2G4* | Bos taurus phospholipase A2 group IVA |
| 75 | *PLCB2* | Bos taurus phospholipase C beta 2 |
| 76 | *PLD1* | Bos taurus phospholipase D1 |
| 77 | *PRKACA* | Bos taurus protein kinase cAMP-activated catalytic subunit alpha |
| 78 | *PRKCA* | Bos taurus protein kinase C alpha |
| 79 | *PTAFR* | Bos taurus platelet activating factor receptor |
| 80 | *PTEN* | Bos taurus phosphatase and tensin homolog |
| 81 | *PTPRC* | Bos taurus protein tyrosine phosphatase, receptor type, C |
| 82 | *RGS4* | Bos taurus regulator of G protein signaling 4 |
| 83 | *RSP9* | Bos taurus ribosomal protein S9 |
| 84 | *SAA3* | Bos taurus serum amyloid A 3 |
| 85 | *SELL* | Bos taurus selectin L |
| 86 | *SLC6A4* | Bos taurus solute carrier family 6 member 4 |
| 87 | *STAT1* | Bos taurus signal transducer and activator of transcription1 |
| 88 | *STAT3* | Bos taurus signal transducer and activator of transcription3 |
| 89 | *STAT5A* | Bos taurus signal transducer and activator of transcription5A |
| 90 | *STAT5B* | Bos taurus signal transducer and activator of transcription 5B |
| 91 | *TGFB* | Bos taurus transforming growth factor beta 1 |
| 92 | *TLR-2* | Bos taurus toll like receptor 2 |
| 93 | *TLR-4* | Bos taurus toll like receptor 4 |
| 94 | *TNF* | Bos taurus tumor necrosis factor |
| 95 | *TPH1* | Bos taurus tryptophan hydroxylase 1 |

**Supplementary Table S2.** Gene expression of 91 tested genes in peripheral leukocytes after a 10-d 5-hydroxytryptophan (5-HTP, 90 mg/d; n = 8), fluoxetine (FLX, 40 mg/d; n = 8) or saline (CON; n = 8) oral supplementation to dairy calves. Data is presented as △Ct (cycle threshold) of each gene using the formula △Ct = Ct – geometric mean of 4 housekeeping genes, and the △△CT (△△CT = △Ct treatment - △Ct control) ± standard error of the mean (SEM). Significance was declared at *P*-value ≤ 0.05 (bolded) and tendencies at 0.05 < *P-*value ≤ 0.10.

|  | **Gene** | **CON**  (△Ct) | **5-HTP**  (△Ct) | **FLX**  (△Ct) | **5-HTP *vs.* CON**  (△△Ct ± SEM) | ***P*-value** | **FLX *vs.* CON**  △△Ct ± SEM | ***P*-value** |
| --- | --- | --- | --- | --- | --- | --- | --- | --- |
| 1 | *5-HT1A* | 3.16 | 2.22 | 2.64 | -0.94 ± 0.34 | **0.02** | -0.52 ± 0.34 | 0.15 |
| 2 | *5-HT1B* | 5.46 | 4.49 | 4.95 | -0.96 ± 0.37 | **0.02** | -0.51 ± 0.37 | 0.18 |
| 3 | *5-HT1D* | 2.54 | 1.64 | 2.03 | -0.90 ± 0.33 | **0.02** | -0.50 ± 0.33 | 0.15 |
| 4 | *5-HT1F* | 3.61 | 2.74 | 3.18 | -0.87 ± 0.39 | **0.04** | -0.43 ± 0.39 | 0.28 |
| 5 | *5-HT2A* | 6.92 | 6.84 | 12.76 | -0.08 ± 1.64 | 0.96 | 5.27 ± 1.64 | **0.01** |
| 6 | *5-HT2B* | -4.02 | -4.71 | -4.45 | -0.69 ± 0.33 | 0.06 | -0.42 ± 0.33 | 0.22 |
| 7 | *5-HT2C* | 9.73 | 8.38 | 8.81 | -1.35 ± 1.47 | 0.13 | -2.06 ± 1.47 | **0.04** |
| 8 | *5-HT3A* | 10.51 | 13.65 | 16.76 | 3.14 ± 2.01 | 0.06 | 6.25 ± 2.01 | **0.001** |
| 9 | *5-HT3B* | 12.03 | 9.28 | 11.18 | -2.75 ± 1.34 | **0.05** | -0.85 ± 1.34 | 0.53 |
| 10 | *5-HT3C* | 0.98 | 0.08 | 0.48 | -0.90 ± 0.32 | **0.01** | -0.50 ± 0.32 | 0.14 |
| 11 | *5-HT4* | 4.79 | 3.86 | 4.13 | -0.94 ± 0.35 | **0.02** | -0.66 ± 0.35 | 0.08 |
| 12 | *5-HT5A* | 6.95 | 6.79 | 7.23 | -0.16 ± -0.63 | 0.81 | 0.28 ± 0.63 | 0.66 |
| 13 | *5-HT6* | 6.24 | 5.50 | 6.18 | -0.74 ± 0.45 | 0.13 | -0.06 ± 0.45 | 0.89 |
| 14 | *5-HT7* | 3.80 | 3.18 | 3.48 | -0.62 ± 0.42 | 0.16 | -0.32 ± 0.42 | 0.46 |
| 15 | *ADCY1* | 7.71 | 7.02 | 7.27 | -0.69 ± 0.40 | **0.03** | -0.45 ± 0.40 | 0.14 |
| 16 | *AKT1* | 3.59 | 2.84 | 2.60 | -0.75 ± 0.29 | **0.02** | -0.82 ± 0.29 | **0.01** |
| 17 | *AKT2* | 3.01 | 2.47 | 2.51 | -0.54 ± 0.28 | 0.08 | -0.50 ± 0.28 | 0.10 |
| 18 | *ALDH2* | -0.10 | -0.37 | -0.43 | -0.27 ± 0.21 | 0.22 | -0.33 ± 0.21 | 0.14 |
| 19 | *APAF1* | 4.07 | 3.79 | 3.68 | -0.28 ± 0.26 | 0.30 | -0.39 ± 0.23 | 0.11 |
| 20 | *BAX* | 3.15 | 3.11 | 3.21 | -0.04 ± 0.15 | 0.79 | 0.06 ± 0.15 | 0.67 |
| 21 | *BCL2* | 4.56 | 4.37 | 4.23 | -0.19 ± 0.32 | 0.57 | -0.33 ± 0.32 | 0.32 |
| 22 | *CASP3* | 5.67 | 6.37 | 5.92 | 0.80 ± 0.74 | 0.36 | 0.25 ± 0.78 | 0.76 |
| 23 | *CASP8* | 4.36 | 4.36 | 3.85 | -0.001 ± 0.27 | 0.99 | -0.51 ± 0.26 | 0.07 |
| 24 | *CASP9* | 4.39 | 4.68 | 4.17 | 0.29 ± 0.38 | 0.46 | -0.22 ± 0.40 | 0.59 |
| 25 | *CCL22* | 16.57 | 14.20 | 14.20 | -2.37 ± 2.13 | 0.29 | -2.36 ± 1,13 | 0.29 |
| 26 | *CCND1* | 14.90 | 14.04 | 10.3 | -0.83 ± 1.70 | 0.63 | -4.61 ± 1.79 | **0.02** |
| 27 | *CD14* | 3.74 | 3.11 | 2.57 | -0.63 ± 0.52 | 0.26 | -1.16 ± 0.53 | **0.05** |
| 28 | *CD19* | 2.22 | 2.01 | 1.90 | -0.21 ± 0.31 | 0.62 | -0.32 ± 0.44 | 0.48 |
| 29 | *CD28* | 5.33 | 5.41 | 6.36 | 0.08 ± 0.60 | 0.90 | 1.03 ± 0.60 | 0.11 |
| 30 | *CD4* | 3.55 | 3.47 | 4.38 | -0.07 ± 0.50 | 0.89 | 0.82 ± 0.50 | 0.12 |
| 31 | *CD44* | -1.18 | -0.87 | -0.95 | 0.31 ± 0.24 | 0.22 | 0.22 ± 0.24 | 0.36 |
| 32 | *CD80* | 3.50 | 2.95 | 2.50 | -0.55 ± 0.31 | 0.10 | -1.00 ± 0.31 | **0.01** |
| 33 | *CD8A* | 7.24 | 7.22 | 6.96 | -0.03 ± 0.22 | 0.90 | -0.28 ± 0.22 | 0.21 |
| 34 | *CD8B* | 5.40 | 5.69 | 5.62 | 0.29 ± 0.46 | 0.54 | 0.22 ± 0.46 | 0.64 |
| 35 | *CREB1* | 1.32 | 1.31 | 1.02 | -0.01 ± 0.32 | 0.97 | -0.30 ± 0.32 | 0.36 |
| 36 | *CTLA4* | 5.87 | 4.83 | 5.30 | -1.04 ± 0.33 | **0.01** | -0.57 ± 0.33 | 0.10 |
| 37 | *CXCL10* | 2.72 | 2.43 | 2.23 | -0.29 ± 0.28 | 0.31 | -0.49 ± 0.28 | 0.09 |
| 38 | *CXCL8* | -1.31 | -0.45 | -0.56 | 0.86 ± 0.59 | 0.17 | 0.75 ± 0.63 | 0.25 |
| 39 | *DDC* | 8.87 | 7.32 | 7.48 | -1.55 ± 0.54 | **0.01** | -1.39 ± 0.57 | **0.03** |
| 40 | *DGKA* | 3.57 | 3.32 | 3.17 | -0.26 ± 0.35 | 0.48 | -0.40 ± 0.35 | 0.28 |
| 41 | *FASLG* | 7.59 | 7.22 | 7.32 | --0.37 ± 1.36 | 0.41 | 0.026 ± 1.35 | 0.85 |
| 42 | *GSK3B* | 2.42 | 2.37 | 2.13 | -0.05 ± 0.27 | 0.85 | -0.30 ± 0.27 | 0.29 |
| 43 | *HP* | 3.17 | 2.86 | 0.98 | -0.31 ± 0.85 | 0.72 | -2.19 ± 0.85 | **0.02** |
| 44 | *IDO1* | 4.05 | 4.06 | 3.38 | 0.01 ± 0.59 | 0.99 | -0.67 ± 0.59 | 0.28 |
| 45 | *IFNG* | 4.15 | 3.26 | 3.65 | -0.88 ± 0.32 | **0.02** | -0.49 ± 0.32 | 0.15 |
| 46 | *IL10* | 5.37 | 4.84 | 4.44 | -0.53 ± 0.71 | 0.47 | -0.93 ± 0.71 | 0.21 |
| 47 | *IL12A* | 14.29 | 12.01 | 12.61 | -2.29 ± 2.33 | 0.34 | -1.68 ± 2.33 | 0.48 |
| 48 | *IL12B* | 5.99 | 5.32 | 4.54 | -0.67 ± 0.61 | 0.29 | -1.45 ± 0.59 | **0.03** |
| 49 | *IL13* | 0.20 | -0.64 | -0.31 | -0.84 ± 0.32 | **0.02** | -0.51 ± 0.32 | 0.14 |
| 50 | *IL17A* | -1.29 | -2.34 | -1.87 | -1.06 ± 0.33 | **0.01** | -0.58 ± 0.33 | 0.10 |
| 51 | *IL1A* | 6.39 | 5.96 | 5.63 | -0.42 ± 0.64 | 0.52 | -0.75 ± 0.64 | 0.26 |
| 52 | *IL1B* | 2.76 | 2.16 | 1.66 | -0.60 ± 0.37 | 0.13 | -1.09 ± 0.37 | **0.01** |
| 53 | *IL2* | 11.36 | 7.24 | 11.07 | -4.13 ± 1.43 | **0.01** | -0.29 ± 1.28 | 0.82 |
| 54 | *IL4* | 7.09 | 5.49 | 6.18 | -1.60 ± 0.32 | **< 0.001** | -0.91 ± 0.31 | **0.01** |
| 55 | *IL6* | 13.72 | 13.75 | 12.33 | 0.03 ± 2.11 | 0.99 | -1.38 ± 2.10 | 0.52 |
| 56 | *LAMP2* | 1.13 | 0.83 | 0.63 | -0.31 ± 0.18 | 0.10 | -0.51 ± 0.16 | **0.01** |
| 57 | *MAOA* | 5.72 | 5.00 | 4.43 | -1.01 ± 0.43 | **0.03** | -1.29 ± 0.43 | **<0.001** |
| 58 | *MAOB* | 9.67 | 7.72 | 7.57 | -1.94 ± 1.08 | 0.09 | -2.10 ± 1.08 | 0.07 |
| 59 | *MAP1LC3A* | 4.19 | 4.07 | 4.30 | -0.12 ± 0.24 | 0.63 | 0.11 ± 0.25 | 0.68 |
| 60 | *MAP1LC3B* | 0.94 | 1.04 | 1.23 | 0.09 ± 0.29 | 0.74 | 0.29 ± 0.29 | 0.34 |
| 61 | *MAP2K6* | 5.00 | 4.31 | 4.65 | -0.69 ± 0.32 | **0.05** | -0.35 ± 0.32 | 0.29 |
| 62 | *MAPK1* | 0.36 | 0.53 | 0.71 | -0.16 ± 0.19 | 0.40 | 0.35 ± 0.19 | 0.09 |
| 63 | *MAPK14* | -1.17 | -0.65 | -0.16 | 0.52 ± 0.32 | 0.13 | 1.00 ± 0.32 | **0.01** |
| 64 | *MAPK3* | 4.47 | 3.81 | 3.65 | -0.66 ± 0.30 | **0.05** | -0.81 ± 0.30 | **0.01** |
| 65 | *MTOR* | 3.86 | 3.67 | 3.52 | -0.19 ± 0.32 | 0.50 | -0.34 ± 0.32 | 0.67 |
| 66 | *NOS2* | 12.25 | 12.79 | 9.80 | 0.54 ± 1.70 | 0.76 | -2.45 ± 1.70 | 0.17 |
| 67 | *PCNA* | 1.68 | 1.56 | 0.98 | -0.12 ± 0.40 | 0.76 | -0.69 ± 0.40 | 0.10 |
| 68 | *PDGFA* | 6.19 | 5.63 | 6.45 | -0.55 ± 0.33 | 0.12 | 0.26 ± 0.34 | 0.47 |
| 69 | *PIK3CB* | 3.04 | 3.06 | 2.37 | 0.02 ± 0.24 | 0.95 | -0.66 ± 0.23 | **0.01** |
| 70 | *PLA2G4* | 2.24 | 2.30 | 2.81 | 0.06 ± 0.37 | 0.88 | 0.57 ± 0.37 | 0.15 |
| 71 | *PLCB2* | 4.04 | 3.30 | 3.30 | -0.74 ± 0.32 | **0.04** | -0.74 ± 0.32 | **0.04** |
| 72 | *PLD1* | 3.16 | 3.26 | 3.16 | -0.10 ± 0.31 | 0.75 | -0.01 ± 0.30 | 0.99 |
| 73 | *PRKACA* | 2.77 | 2.32 | 2.14 | -0.45 ± 0.26 | 0.11 | -0.63 ± 0.26 | **0.01** |
| 74 | *PRKCA* | 9.30 | 8.10 | 8.28 | -1.20 ± 0.84 | 0.18 | -1.02 ± 0.85 | 0.25 |
| 75 | *PTAFR* | 1.27 | 1.21 | 1.60 | -0.06 ± 0.35 | 0.63 | 0.32 ± 0.35 | 0.97 |
| 76 | *PTEN* | -0.07 | -0.87 | -0.48 | -0.79 ± 0.24 | **0.01** | -0.41 ± 0.24 | 0.11 |
| 77 | *PTPRC* | -0.68 | -0.85 | -1.39 | -0.17 ± 0.41 | 0.69 | -0.70 ± 0.41 | 0.11 |
| 78 | *REL* | 1.40 | 1.23 | 0.58 | -0.17 ± 0.43 | 0.70 | -0.83 ± 0.43 | 0.07 |
| 79 | *RGS4* | 4.99 | 6.20 | 4.67 | 1.20 ± 1.36 | 0.39 | -0.33 ± 1.36 | 0.81 |
| 80 | *SAA3* | 4.16 | 3.49 | 3.69 | -0.67 ± 0.33 | 0.06 | -0.47 ± 0.33 | 0.19 |
| 81 | *SELL* | 0.46 | -0.05 | -0.16 | -0.50 ± 0.30 | 0.12 | -0.62 ± 0.30 | 0.06 |
| 82 | *SLC6A4* | 1.17 | 1.21 | 2.18 | 0.04 ± 0.62 | 0.94 | 1.00 ± 0.62 | 0.13 |
| 83 | *STAT1* | 1.07 | 0.92 | 0.22 | -0.16 ± 0.33 | 0.65 | -0.85 ± 0.33 | **0.02** |
| 84 | *STAT3* | 2.79 | 2.59 | 2.20 | -0.20 ± 0.29 | 0.43 | -0.59 ± 0.29 | 0.16 |
| 85 | *STAT5A* | 5.75 | 5.13 | 5.22 | -0.61 ± 0.37 | 0.12 | -0.53 ± 0.18 | 0.18 |
| 86 | *STAT5B* | 3.50 | 2.95 | 2.72 | -0.55 ± 0.31 | 0.10 | 0.78 ± 0.29 | **0.02** |
| 87 | *TGFB* | -1.40 | -1.13 | -0.56 | 0.27 ± 0.37 | 0.48 | 0.84 ± 0.37 | **0.04** |
| 88 | *TLR2* | 3.58 | 3.10 | 3.33 | -0.50 ± 0.31 | 0.14 | -0.25 ± 0.31 | 0.44 |
| 89 | *TLR4* | 4.56 | 3.92 | 3.57 | -0.64 ± 0.40 | 0.14 | -0.98 ± 0.40 | **0.03** |
| 90 | *TNF* | 4.60 | 4.63 | 4.23 | 0.03 ± 0.29 | 0.88 | -0.37 ± 0.22 | 0.10 |
| 91 | *TPH1* | 6.14 | 6.14 | 7.04 | -0.002 ± 0.64 | 0.97 | 0.89 ± 0.64 | 0.18 |
